# Influence of body mass index and periprostatic fat on rectal dosimetry in permanent seed prostate brachytherapy

**DOI:** 10.1186/1748-717X-9-93

**Published:** 2014-04-14

**Authors:** David Tiberi, Nelson Gruszczynski, Aliza Meissner, Guila Delouya, Daniel Taussky

**Affiliations:** 1 Département de Radio-Oncologie, Centre Hospitalier de l’Université de Montréal (CHUM) - Hôpital Notre-Dame, Montréal, Québec, Canada; 2 Centre Hospitalier de l’Université de Montréal (CHUM) – Hôpital Notre-Dame, Department of Radiation Oncology, 1560 rue Sherbrooke E, Montréal, Québec H2L 4 M1, Canada

**Keywords:** Prostate cancer, Adipose tissue, Body mass index, Brachytherapy, Rectal dose

## Abstract

**Purpose:**

We examined the influence of body mass index (BMI) and body fat distribution on rectal dose in patients treated with permanent seed brachytherapy for localized prostate cancer.

**Methods and materials:**

We analyzed 213 patients treated with I^125^ seed brachytherapy for localized prostate cancer. BMI and rectal dosimetry data for all patients were available. Data on visceral and subcutaneous fat distribution at the level of the iliac crest (n = 140) as well as the distribution of periprostatic and subcutaneous fat at the symphysis pubis level were obtained (n = 117). Fat distribution was manually contoured on CT on day 30 after brachytherapy. The correlation between BMI, fat distribution and rectal dose (R100 (in cc), R150 (cc), D2 (Gy)) was analyzed using the Spearman correlation coefficient. Differences in rectal dose between tertiles of body fat distribution were calculated using nonparametric tests.

**Results:**

Periprostatic adipose was only weakly correlated with BMI (r = 0.0.245, p = 0.008) and only weakly correlated with the other fat measurements (r = 0.31-0.37, p < 0.001). On the other hand, BMI was correlated with all other fat measurements (≥0.58, p < 0.001). All the other fat measurements were strongly correlated with each other (r = 0.5-0.87, p < 0.001). Patients with an R100 of >1.3 cc (23% of patients) had less visceral fat (p = 0.004), less subcutaneous fat at the level of the iliac crest (p = 0.046) and a lower BMI (26.8 kg/m^2^ vs. 28.5 kg/m^2^, p = 0.02) than patients with an R100 of <1.3 cc. Results were very similar when comparing an R100 of >1.0 cc (34% of patients) across the tertiles. None of the tested linear regression models were predictive (max 12%) of dose to the rectum.

**Conclusion:**

Dose to the rectum is dependent on BMI and body fat distribution. Periprostatic fat does not influence rectal dose. Dose to the rectum remains difficult to predict and depends on many factors, one of which is body fat distribution.

## Background

Several studies have shown a dose-volume dependence on rectal bleeding after permanent seed brachytherapy (BT) [1-4]. Consequently, several European societies have published guidelines regarding the rectal dose [5].

Currently, little is known about the factors that might influence the dose to the rectum in BT. Some studies have shown that rectal dose decreases with the physician’s clinical experience [6] while others found no such effect. Certain groups [7] found that the dose to the rectum increased with prostate volume [8]. Recently, Patil et al. [9] identified body mass index (BMI) as one of several factors that are negatively correlated with rectal dosimetry. It is clear that little consensus exists regarding which variables have an impact on the rectal dose in BT.

BMI has often been used as a surrogate for adipose tissue. We hypothesized that directly measuring adipose tissue distribution, especially around the prostate, would more accurately predict the dose to the rectum than using BMI alone.

## Methods

Our cohort consisted of both low-risk patients (stage T1c-T2a, PSA level ≤10 ng/mL and Gleason score ≤6) and lower tier intermediate risk patients (T2b or PSA level 10-20 ng/mL or Gleason 7 in ≤30% of biopsies) as defined by the 2010 American Joint Committee on Cancer (AJCC) clinical stage guidelines [10]. The patients had no history of prior hormonal therapy and were exclusively treated with permanent iodine-125 seed brachytherapy to 144Gy (BT) with an intraoperative planning system.

Adipose tissue measurements were available for patients who were part of two prior studies in our center investigating the influence of adipose tissue distribution on PSA bounce after BT [11,12]. The BMI was calculated for all these patients. Height and weight data were obtained from preoperative records.

Adipose tissue distribution was measured on computed tomography (CT) scan images obtained 30 days following BT.

**Figure 1:**
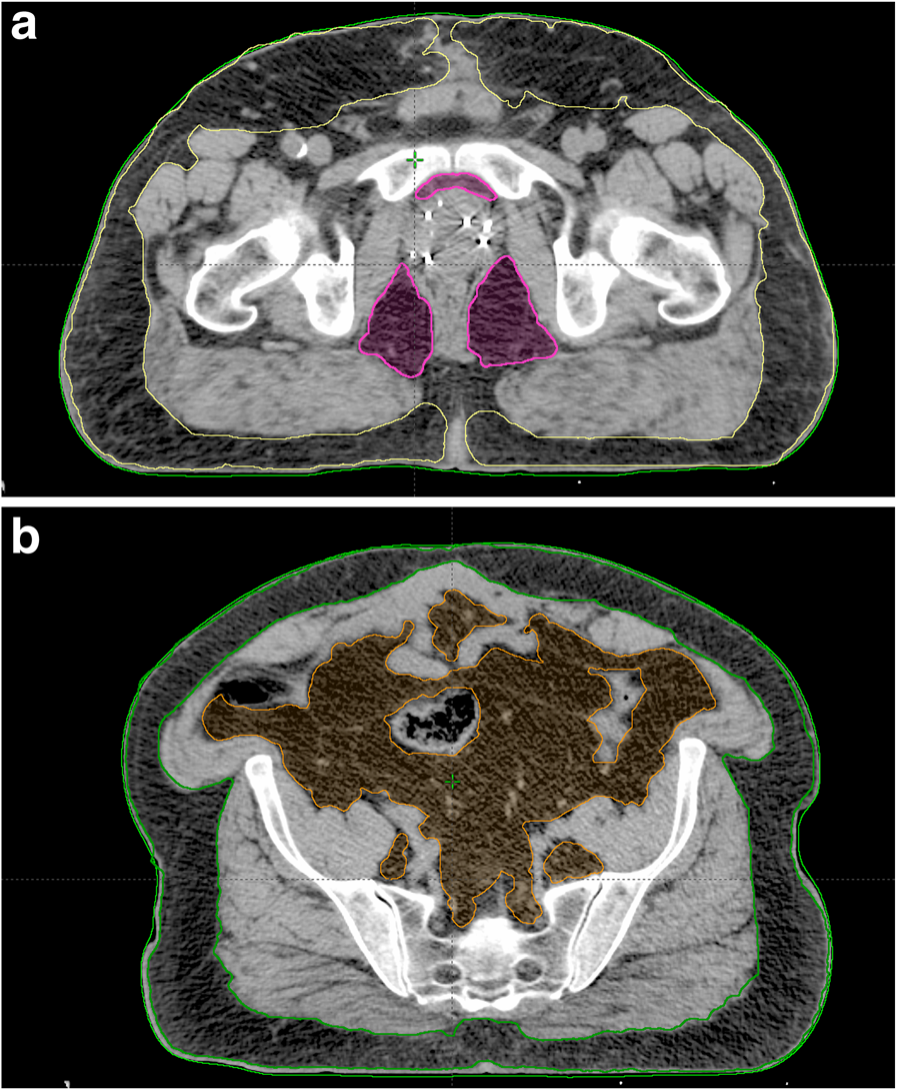
**Adipose tissue measurements (a) periprostatic fat (pink), and subcutaneous fatat symphysis pubis level (yellow).(b)** abdominal visceral fat (orange) and subcutaneous adipose tissue at the level of the iliac crest (green).

All adipose tissue was contoured manually using the Eclipse treatment planning system (Varian Medical Systems, Inc., Palo Alto, CA) on three consecutive 3 mm slices. Periprostatic adipose tissue was contoured anteriorly and posteriorly to the prostate from the level of the superior border of the symphysis onwards in the caudal direction (Figure 1a). Unlike other studies that included the tissue medial to the levator ani muscles in their calculation of periprostatic fat [13], we chose to exclude this area since it is dependent on rectal filling and thus highly variable. Subcutaneous adipose tissue at the symphysis level was contoured from the upper level of the pubic symphysis onwards in the caudal direction on three 3-mm slices (Figure 1a). Visceral fat and again the subcutaneous fat were measured from the iliac crest onwards in the caudal direction on three slices that often corresponds to the 4^th^ lumbar vertebra (Figure 1b).

Most patients with fat measurements were part of two studies investigating the influence of fat distribution on PSA-bounce. However, only one study (n = 54) [11] included measurement of periprostatic fat in addition to the other measurements. The other study (n = 68) [12] measured most of the other fat distributions. We then randomly added other patients from our database that received permanent seed brachytherapy and measured their fat distribution and the BMI. All patients with fat measurements also had BMI data. BMI was available for 211 patients, perirpostatic fat in 116, subcutaneous fat at the symphysis level in 113 and at the iliac crest level in 136 patients and visceral fat in 139 patients.

All patients with fat measurements also had rectal dosimetry available. Rectal dosimetry was measured on CT scan images obtained 30 days following brachytherapy. The anterior rectum wall was contoured on all slices where prostate was visible. Dose-volume points analyzed were the volume in cubic centimeters of the anterior rectal wall receiving 100% and 150% of the prescribed dose (R100 and R150 in cc) and the isodose that encompassed 2% of the rectal volume (D2 in Gy). This retrospective analysis was approved by the institutional review board of the Centre Hospitalier de l’Université de Montréal (CHUM), approval number 12.240

### Statistical methods

The correlation between BMI, adipose tissue distribution, clinical factors and rectum dosimetry was analyzed using the Spearman correlation coefficient.

Differences in rectal dose between tertiles of body fat distribution were calculated using nonparametric tests, either the Kruskal Wallis or the Chi Square test when appropriate.

A linear regression was used for the multivariate analysis. Factors with a p < 0.1 in the correlation analysis were included in this multivariate analysis. All tests were two sided. Associations or differences of *p* <0.05 were deemed significant. All the data was analyzed using SPSS 20.0 for Windows (IBM SPSS, IBM Corp., Armonk, NY).

## Results

Clinical patient characteristics are listed in Table 1. Periprostatic adipose tissue seems to be independent of other fat measurements and BMI. It was only weakly correlated with BMI (r = 0.245, p = 0.008) and only weakly correlated with the other fat measurements (r = 0.31-0.37, p < 0.001), while BMI was correlated with all other fat measurements (≥0.58, p < 0.001) and the other fat measurements were strongly correlated with each other (r = 0.5-0.87, p < 0.001) (Table 2).

**Table 1:** Clinical, implant and dosimetric characteristics

**Parameter**	**Mean/median (SD)**
BMI	28.1/28.0 (4.5)
Activity (mCi)	0.48/0.44 (range 0.32-0.68)
Prostate volume (cc)	36.2 (11.6)
Prostate V100 (%)	96.5/94.9 (5.7)
Prostate V150 (%)	62.8/63.5 (14.0)
Prostate D90 (Gy) <130 Gy	159.6/158.0 (26.3) 14.7%
Rectum R100 (cc)	0.80/0.62 (0.9)
Rectum R150 (cc)	0.19/0.08 (0.6)
Rectum D2 (Gy)	212/208 (96.8)
Age (y)	68.2/69 (6.4)
PSA (mg/mL) PSA <10 (mg/mL)	6.26/5.8 (3.3) 92%
Gleason 6/7	69.2%/30.8%
T1 (AJCC 1997) T2	69% 31%

D90: minimum dose received by 90% of prostate volume at Day 30.

V100: prostate volume receiving 100% of the prescribed dose at Day 30.

R100/R150: volume of anterior rectal wall receiving 100%/150% of the prescribed dose in cc.

D2: isodose that encompassed 2% of the rectal volume in Gy.

**Table 2:** Correlation between body mass index (BMI) and adipose fat distribution

**Factor**	**Median (range)**	**Correlation with BMI**	
		**R =**	**P=**
Subcutaneous fat (iliac crest)	115.6 (26.06-315.5)	0.69	<0.001
Subcutaneous fat (symphysis)	123.7 (43.7-322.5)	0.687	<0.001
Visceral fat	69.1 (16.4-184.3)	0.599	<0.001
Periprostatic fat	13.4 (1.26-34.08)	0.245	0.008

All body fat measurements were weakly (range correlation coeff. -0.19 to –0.29) but significantly (p = 0.02 to <0.001) inversely correlated with the dose to the rectum, except for periprostatic fat. (p = 0.38-0.83 for the D2, V100 and V150 of the rectum).

**Figure 2:**
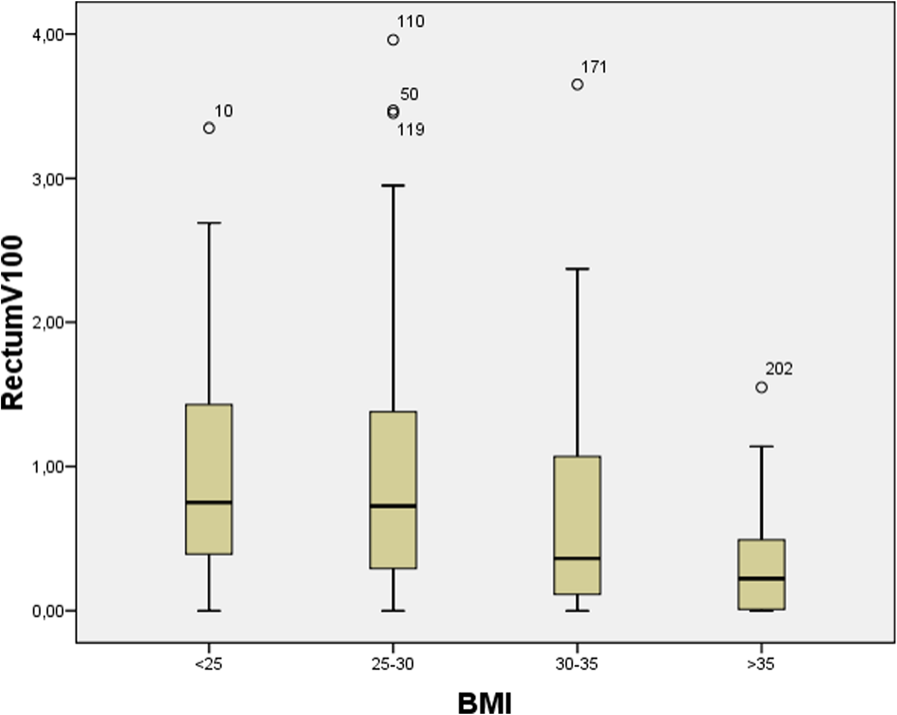
**Box plot of V100 of rectum dosimetry stratified by BMI.** R100: volume of anterior rectal wall receiving 100% of the prescribed dose in cc. The middle line in the box is the median. The length of box is the interquartile range (IQR). The “o” symbols indicate values more than 1.5 IQR’s but less than 3 IQR’s. Abbreviations: BMI; body mass index.

**Figure 3:**
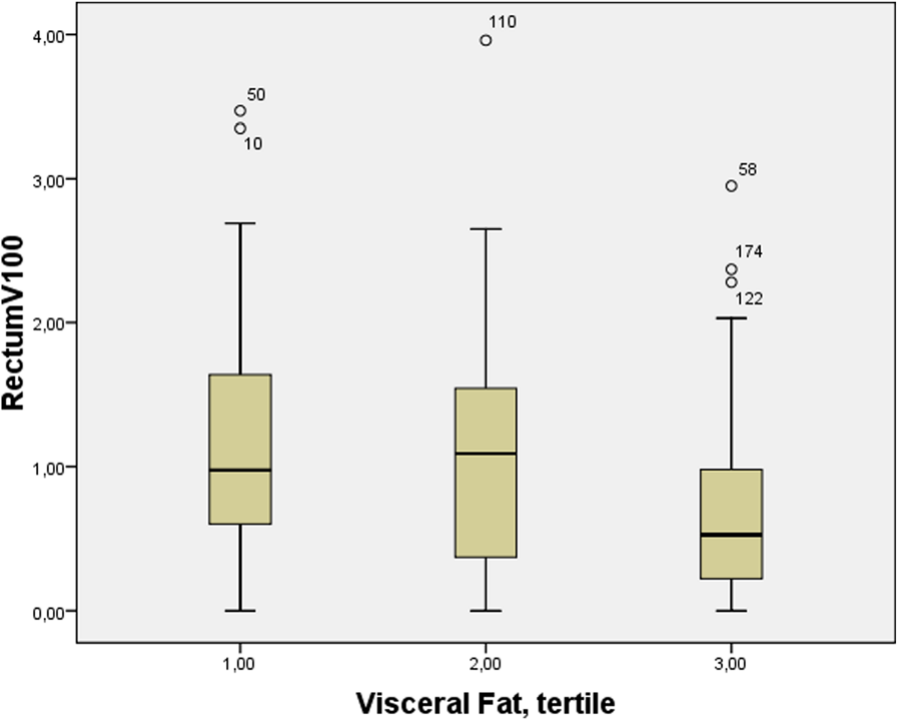
**Box plot of V100 of rectum dosimetry stratified by tertiles of visceral fat R100: volume of anterior rectal wall receiving 100% of the prescribed dose in cc.** The middle line in the box is the median. The length of box is the interquartile range (IQR). The “o” symbols indicate values more than 1.5 IQR’s but less than 3 IQR’s. Abbreviations: BMI; body mass index.

34% of patients had an R100 of >1.0 cc and 23% had an R100 of >1.3 cc. Compared to patients with an R100 of <1.3 cc, patients with the clinically significant R100 of >1.3 cc had significantly less (p = 0.004) visceral fat (mean 63 cc, SD 27 cc vs. 78 cc, SD 33), less subcutaneous fat at the level of the iliac crest (117 cc SD41 vs. 138 cc SD 52, p = 0.046) and a lower BMI (26.8 kg/m^2^ vs. 28.5 kg/m^2^, p = 0.02). Table 3 lists the R100 of the rectum according to fat tertiles of the different adipose tissues and BMI. When grouped according to the different BMI categories, patients who were normal weight (≤25 kg/m^2^) overweight (25-30 kg/m^2^), obese (30-35 kg/m^2^) or morbidly obese (>35 kg/m^2^) were not more like likely to have an R100 > 1.3 cc (p = 0.2 Chi Square test). See Figure 2 for a stratification of rectum dosimetry according to BMI and Figure 3 for stratification according to tertile of visceral fat.

**Table 3:** Rectum dosimetry according to tertiles of adipose tissue measurement

	**R100**	P=*	**R150**	**P=**	**D2**	**P=**
Visceral fat		**0.003**		**0.004**		**0.013**
1^st^ Tertile	1.2/0.98 (0.8)		0.17/0.36 (0.4)		272/257 (108)	
2^nd^ Tertile	1.1/1.1 (0.8)		0.33/0.23 (0.4)		255/244 (87)	
3^rd^ Tertile	0.7/0.5 (0.7)		0.16/0.07 (0.3)		213/204 (96)	
Subcut. fat (iliac crest)		**0.004**		**0.01**		**0.017**
1^st^ Tertile	1.2/0.98 (0.9)		0.33/0.17 (0.4)		274/257 (112)	
2^nd^ Tertile	1.0/1.0 (0.7)		0.3/0.2 (0.3)		251/241 (85)	
3^rd^ Tertile	0.7/0.5 (0.7)		0.17/0.07 (0.3)		216/205 (94)	
Subcut. fat (symphysis)		0.09		0.14		0.23
1^st^ Tertile	1.1/0.8 (0.9)		0.26/0.11 (0.4)		260/244 (119)	
2^nd^ Tertile	1.0/1.0 (0.7)		0.29/0.14 (0.3)		242/230 (87)	
3^rd^ Tertile	0.70/0.52 (0.6)		0.17/0.06 (0.2)		217/207 (94)	
Periprostatic Fat		0.86		0.55		0.61
1^st^ Tertile	0.96/0.72 (0.9)		0.25/0.16 (0.4)		238/204 (131)	
2^nd^ Tertile	0.93/0.89 (0.7)		0.24/0.16 (0.3)		238/239 (78)	
3^rd^ Tertile	0.86/0.73 (0.7)		0.24/0.08 (0.3)		243/226 (107)	
BMI		**0.001**		**0.003**		**<0.001**
<25 kg/m^2^ (n = 43)	0.96/0.75 (0.8)		0.23/0.08 (0.3)		239/221 (110)	
25-30 kg/m^2^ (n = 98)	0.98/0.73 (0.9)		0.28/0.12 (0.4)		239/227 (90)	
30-35 kg/m^2^ (n = 53)	0.65/0.36 (0.7)		0.26/0.03 (0.8)		191/178 (92)	
>35 kg/m^2^ (n = 17)	0.38/0.22 (0.5)		0.06/0.01 (0.1)		156/157 (75)	

Results are in mean/median (SD). Differences between tertiles were calculated using the Kruskal Wallis nonparametric test. *Bold values indicate a significant result

R100/R150: volume of anterior rectal wall receiving 100%/150% of the prescribed dose in cc.

D2: isodose that encompassed 2% of the rectal volume in Gy.

Results were very similar when comparing an R100 of <1.0 cc across the tertiles of visceral fat (p = 0.02), subcutaneous fat at the level of the iliac crest (p = 0.01), periprostatic fat (p = 0.7) and subcutaneous fat at the symphysis level (p = 0.2).

Table 3 lists rectum dosimetry according to tertile of fat tissue measurement. Dosimetry to the rectum decreases significantly over all 3 tertiles for visceral fat, subcutaneous fat (iliac crest level) and the 4 BMI categories but not for periprostatic fat or subcutaneous fat (symphysis level).

Different linear regression models were then tested in a multivariate analysis to predict the dose to the rectum (R100, R150 and D2) as a continuous variable. Most models had a very small Adjusted R square value (max 0.12). Therefore these models have very little predictive (max 12%) value and we decided not to report them in this paper.

It is important to mention that none of the fat tissue measurements were correlated to prostate volume, age, Gleason score or prostate specific antigen (PSA). BMI was only weakly correlated to age (r = -0.16, p = 0.02).

## Discussion

Rectal bleeding following prostate brachytherapy occurs in less than 10% of patients [2,14]. Known risk factors for rectal bleeding are the addition of EBRT [14], age [15] and dose to the rectum. Tran et al. [4] reported a much higher dose R100 for patients with rectal bleeding compared to patients without rectal bleeding (2.3 cc vs. 0.76 cc). Keyes et al. [2] found that patients without any rectal toxicity had a mean R100 of 0.96 cc while those with grade 1-2 rectal toxicity had an R100 ≥ 1.3 cc.

Physicians practicing brachytherapy realize that the dose to the rectum is very difficult to predict intraoperatively even though care is taken to keep it as low as possible. To our knowledge, this is the first report to measure adipose tissue distribution and its influence on rectum dosimetry. Our interest in this subject was awakened by our previous research investigating the influence of BMI and adipose tissue distribution on PSA-bounce [11,16] and the findings of Patil et al. [9], which showed that a higher BMI was associated with a lower dose to the rectum. This finding is not surprising, as it seems intuitive that fatty tissue between the rectum and the prostate acts as a radiation buffer.

We can confirm that BMI in general and fat distribution have an influence on the dose to the rectum. We found that patients with a higher BMI and greater visceral and subcutaneous fat content at the iliac crest level have lower doses to the rectum. As the linear regression analysis found that the various fat measurements and BMI were only weakly predictive of the dose to the rectum, body fat remains only one of the many variables to consider. Rectal filling, another important factor influencing the rectal dose, was not measured in this study. As rectal filling can change from day to day, it is an unreliable variable affecting the dose to the rectum. One can argue that measuring the distance between the rectum and prostate at the time of seed implantation might be a better predictor of the rectal dose. We chose not to use this measurement as we feel this distance is dependent on how deep and at what angle the trans-rectal ultrasound (TRUS) probe is inserted.

Another factor to consider is the timing of the dosimetric evaluation, as it was have previously described that less prostatic edema leads to a higher dose [17].

We decided to directly measure adipose tissue in various regions and specifically around the prostate to see whether this would be a better predictor of dose to the rectum than the BMI. We expected the periprostatic fat to be predictive of dose to the rectum. Surprisingly, the periprostatic fat was the least correlated of all the fat measurements to the received rectal dose. A simple explanation may be that the adipose tissue between the rectum and prostate is compressed during the procedure. However, this compression is relieved by the time of the evaluation of the implant, 30 days after the procedure. At this point, the dose to the rectum should be lower for patients with a large fat pad. Patil et al. [9] and our present study are so far the first to report an influence of BMI on rectal dose in prostate brachytherapy. Their RV100 was very similar to ours with 0.79 cc but with a smaller standard deviation (0.49 versus 0.89). Interestingly, they reported a very similar mean BMI (27.8 kg/m^2^ ± 4.2) compared to our cohort (28.0 kg/m^2^ ± 4.5).

We also found that BMI was itself strongly correlated to all adipose tissue measurements except for periprostatic adipose tissue. Therefore, we believe that measurement of adipose tissue distribution has no advantage over measurement of the BMI to predict dose to the rectum.

We believe that investigation of the periprostatic adipose tissue is an interesting and nascent field. While exploring ways to measure the periprostatic adipose tissue and reviewing the literature, we realized the importance of guidelines in this new research field. Current practices for determining periprostatic adipose tissue vary greatly. One center mentioned that it used in-house automatic software, which included tissue in the levator ani sling [13]. We believe that including the tissue within the sling induces a large bias as the volume depends largely on rectum filling. Bhindi et al. [16] used the diameter of the anterior fat pad as a surrogate, but there is also adipose tissue posterior and lateral to the prostate. Since part of this volume depends on rectum filling, we decided to include the anterior fat pad as well as the fat lateral and posterior to the prostate, but only outside of the levator ani sling.

Ribeiro et al. have shown an influence of periprostatic adipose tissue on prostate cancer aggressiveness in vitro [18]. While all adipose tissue may produce soluble mediators, it may be the local mediators such as IL-6 regulated pathways released by the periprostatic adipose tissue that ultimately have an impact on prostate volume and prostate cancer pathogenesis [19].

## Conclusions

Specific adipose tissue measurements are not superior to BMI in predicting dose to the rectum in permanent seed brachytherapy. Periprostatic adipose tissue is not predictive of dose to the rectum. We need to continue to identify potential factors that may predict the dose to the rectum.

## Competing interest

There is no competing interest concerning the authors.

## Authors’ contributions

DTi, NG, AM, DTa acquired the data, DTa analyzed the data, DTi, GD, DTa drafted the manuscript. All authors read and approved the final manuscript.
